# Primary biliary cholangitis presenting as acute ischemic stroke: A rare association

**DOI:** 10.1002/ccr3.2590

**Published:** 2020-01-04

**Authors:** Bumsoo Park, Sumaiya Islam, Raghavendra C. Vemulapalli, Maria E. Shreve

**Affiliations:** ^1^ Departments of Family Medicine and Urology University of Michigan Medical School Ann Arbor MI USA; ^2^ Department of Family Medicine Henry Ford Health System Wayne State University School of Medicine Detroit MI USA

**Keywords:** hypercholesterolemia, hyponatremia, primary biliary cholangitis, stroke

## Abstract

Primary biliary cholangitis is associated with hyperlipidemia, but studies show that the condition does not increase cardiovascular risks. The case presents acute ischemic stroke with no underlying risk factors and subsequent new diagnosis of primary biliary cholangitis, which can suggest possible association between primary biliary cholangitis and acute stroke.

## Graphical abstract

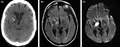

## 1 INTRODUCTION

Primary biliary cholangitis (PBC), formerly known as primary biliary cirrhosis, is an autoimmune inflammatory disorder of the liver which causes progressive destruction of small bile ducts. The disease is rare with an incidence of 4.9 cases per 100 000 person‐years.^1^ PBC mostly affects middle‐aged women, and many cases (20%‐60%) are asymptomatic with incidental diagnosis due to abnormal laboratory findings. The most common presenting symptoms are fatigue and pruritus.^2^ As hyperlipidemia is a common laboratory finding along with the abnormal liver profile, PBC has been hypothesized to increase the risk of cardiovascular and atherosclerotic complications. However, several studies have shown that PBC is not associated with increased risk of cardiovascular events, especially stroke.^3^, ^4^, ^5^, ^6^ We report a case of a woman who presented with acute ischemic stroke and subsequent diagnosis of PBC. The patient demonstrated hyponatremia secondary to extreme hypercholesterolemia with significantly elevated high‐density lipoprotein cholesterol (HDL‐C) levels, an unusual presentation of PBC. To our knowledge, no other cases of acute stroke and/or extremely high HDL‐C have been associated with PBC.

## 2 CASE PRESENTATION

**Figure 1:**
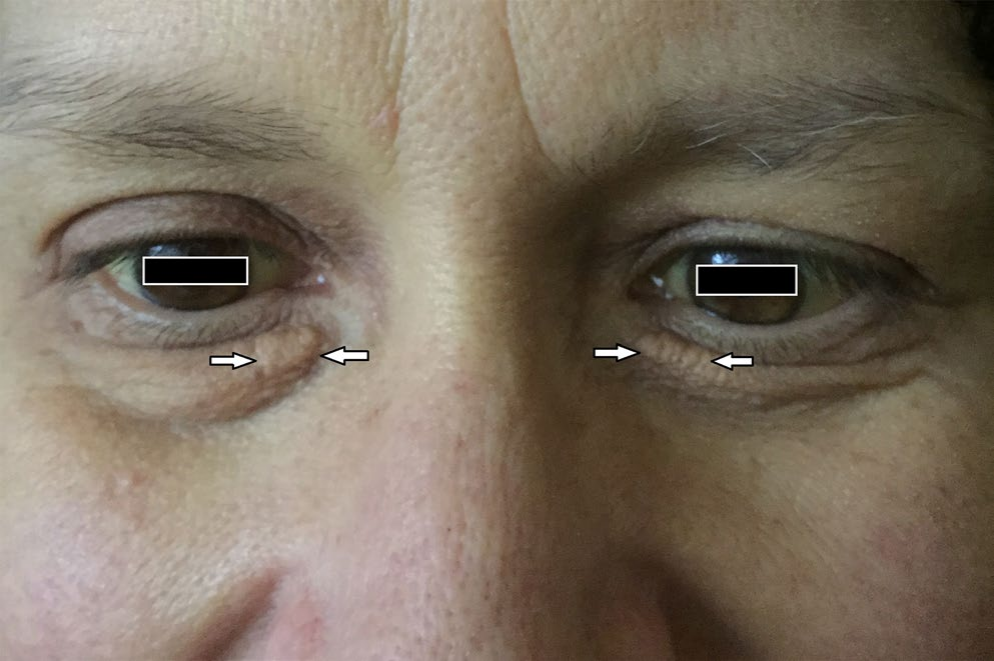
Picture of the patient's face. It reveals bilateral scleral icterus and xanthelasmas on the medial side of bilateral lower eyelids (arrows). This picture is used under permission with a direct written consent from the patient

**Figure 2:**
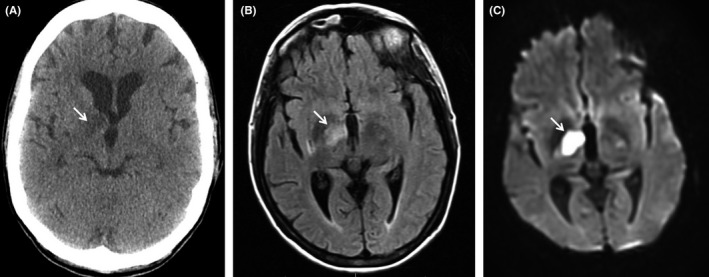
Radiologic studies of the brain. (A) Computed tomography scan of the brain showing 1.5‐cm lucency involving the right thalamus, which potentially suggests acute infarct as one of the differentials. (B) T1‐weighted magnetic resonance imaging that shows increased focal density involving right thalamic area. (C) Diffusion‐weighted magnetic resonance imaging which demonstrates an area of acute infarct in the right thalamus measuring approximately 2.1 × 1.3 cm^2^ in the greatest dimension

A 52‐year‐old woman with no known chronic medical conditions presented to the emergency department with left lower extremity weakness, left facial droop, and slurred speech for 3 days. The patient also reported diffuse body pruritus, yellow skin, and dark urine for 7 days. She denied any history of stroke or other cardiovascular diseases, diabetes, hyperlipidemia, liver disease, exposure to hepatitis, alcohol abuse, or intravenous drug use. She had a family history of lupus in her maternal grandmother and two aunts. Body mass index was 26.63 kg/m^2^. Physical examination was remarkable for scleral icterus and xanthelasma of bilateral medial lower eyelids (Figure 1). Neurological examination demonstrated left facial weakness, decreased strength (4 of 5) of left upper and lower extremities, and decreased rapid repetitive movement with ataxic finger‐to‐nose testing on the left side. Laboratory tests were remarkable for increased aspartate aminotransferase (AST) (93 IU/L), alanine aminotransferase (ALT) (115 IU/L), total bilirubin (10.4 mg/dL), direct bilirubin (5.0 mg/dL), and alkaline phosphatase (ALP) (838 IU/L). Computed tomography (CT) of the head and subsequent magnetic resonance imaging of the brain both revealed acute thalamic infarction of the right side with no evidence of hemorrhagic stroke (Figure 2). Ultrasonography of the abdomen showed mild splenomegaly with no evidence of gallstones or biliary dilatation. CT of the abdomen showed no evidence of biliary obstruction or active hepatocellular disease except incidentally found hepatic hemangioma. The patient was admitted to the hospital and started on aspirin 81 mg and rosuvastatin 20 mg for acute thalamic stroke management. Bilateral carotid duplex test showed 1%‐19% stenosis of left proximal internal carotid artery (ICA) and right bulb, but no stenosis in right ICA or left bulb. Echocardiogram showed no evidence of intracardiac shunts. The patient's neurological symptoms subsequently resolved with minimal residual deficits.

Further workup was performed for hepatic manifestations. Magnetic resonance cholangiopancreatography showed patent intrahepatic and extrahepatic bile ducts including common bile duct with no evidence of focal stricture or filling defect. Antimitochondrial antibody titer was high at >1:320. Based on the diagnostic criteria from the American Association for the Study of Liver Diseases,^7^ PBC was diagnosed. The patient was started on ursodiol 3.25 mg/kg three times daily.

During the hospital course, the patient's serum sodium remained low at around 119 mmol/L. Lipid profile demonstrated extremely high total cholesterol at 2018 mg/dL, extremely high HDL‐C at >200 mg/dL (low‐density lipoprotein‐C [LDL‐C] not calculated), and increased triglycerides at 319 mg/dL. Corrected serum sodium level reflecting hypercholesterolemia using the equation developed by Dimeski et al^8^ was 124 mg/dL. Serum osmolality (293 mOsm/kg) and urine sodium (68 mmol/L) were normal, and urine osmolality (233 mOsm/kg) was slightly decreased. The hyponatremia was thought to be pseudohyponatremia secondary to extreme hypercholesterolemia. The patient was started on rosuvastatin 20 mg, fenofibrate 145 mg, and ezetimibe 10 mg for hypercholesterolemia and was discharged.

The patient is currently under evaluation for liver transplantation with no evidence of decompensated liver failure. Patient has finished physical therapy and speech therapy sessions since the stroke and shows functional improvement. She has been taking ursodiol 300 mg three times daily and rosuvastatin 10 mg once daily. Her most recent liver profiles have improved with AST 34 IU/L, ALT 38 IU/L, total bilirubin 1.0 mg/dL, and ALP 181 IU/L. Her total cholesterol and serum sodium also improved at 267 mg/dL and 140 mg/dL, respectively. LDL‐C and HDL‐C became 179 mg/dL and 68 mg/dL, respectively.

## 3 DISCUSSION

Our patient with PBC presented with acute thalamic stroke, hypercholesterolemia with extremely high HDL‐C levels, and possibly pseudohyponatremia. Studies have concluded that PBC has no significant association with circulatory system‐related mortality^9^ or increased risk of myocardial infarction, stroke, or transient ischemic attack.^4^, ^6^ Our case shows that further longitudinal research is needed regarding stroke risk and PBC.

Hypercholesterolemia causes atherosclerosis through endothelial injury and dysfunction from inflammatory cytokines and oxidation of LDL particles. As high cholesterol in PBC is primarily due to increased lipoprotein‐X which is an abnormal LDL particle that inhibits oxidation of normal LDL particles, it has been hypothesized that hyperlipidemia in PBC might have a protective effect for atherosclerosis.^10^, ^11^ Floreani et al supported this hypothesis by suggesting that increased adiponectin levels in PBC patients might work as a protective factor against atherosclerosis.^12^ However, a systematic review showed that PBC significantly increases the risk of CAD.^13^ Our patient had extreme hypercholesterolemia with very high HDL‐C levels. Three possible mechanisms have been suggested by which PBC causes hypercholesterolemia. First, diminished bile acid synthesis due to cholestasis in PBC downregulates hepatic cholesterol synthesis. This leads to progressive decline in LDL receptors, which subsequently can increase serum LDL cholesterol. Second, PBC causes decreased intestinal cholesterol absorption, which can result in decreased bile acid synthesis and subsequent poor micellar solubilization. Third, cholestasis largely results in increased serum lipoprotein‐X which can originate from either reflux of biliary lipids into plasma or the accumulation of phospholipid and free cholesterol in serum due to reduced lecithin:cholesterol acyltransferase activity.^11^ As the hyperlipidemia in PBC mostly comes from increased lipoprotein‐X which is an LDL derivative, most of the case reports regarding PBC and hypercholesterolemia showed normal or slightly elevated HDL‐C levels.^14^, ^15^, ^16^, ^17^, ^18^ Interestingly, our case shows extremely elevated HDL‐C which presented with acute stroke. It has been well established that HDL‐C level is inversely associated with cardiovascular events. However, recent studies show that extremely high HDL‐C can cause adverse results. Madsen et al found U‐shaped association between HDL‐C level and all‐cause mortality and concluded that extremely high HDL‐C paradoxically increases all‐cause mortality.^19^ Hirata et al also demonstrated association of extremely high HDL‐C with increased atherosclerotic cardiovascular disease (including CAD and stroke) mortality.^20^ Although there is no clear explanation for the association of extremely high HDL‐C and cardiovascular mortality, investigators suggested a possible genetic variant such as cholesterylester transfer protein (CETP) gene mutation. CETP gene is known to be responsible for HDL‐C metabolism, and its polymorphism has been suggested to be associated with accelerated atherosclerosis and cardiovascular risk.^21^ As our patient developed acute ischemic stroke with no underlying risk factors but extremely high HDL‐C, further studies regarding the association of extremely high HDL‐C and cardiovascular risks are needed.

Our patient also demonstrated significant hyponatremia, which was thought to be pseudohyponatremia in the setting of extreme hypercholesterolemia. In marked hyperlipidemia, the sodium concentration per unit of plasma is reduced because the water fraction falls below 80% even though the total sodium concentration in the water phase is unchanged.^22^

A limitation of our report was the lack of equipment to perform lipoprotein electrophoresis, which would have helped to delineate what proportion of lipoprotein was significantly increased, especially with regard to lipoprotein‐X.

In conclusion, our case represents possibly the first report of a patient with newly diagnosed PBC presenting with acute ischemic stroke with no other risk factors of atherosclerosis. Even though studies have shown that PBC does not increase the risk of atherosclerotic complications, we suggest appropriate monitoring, counseling, or appropriate treatment for possible stroke when caring for patients with PBC. Additional studies will be needed regarding the association of extremely high HDL‐C and the risk of cardiovascular complications.

## CONFLICT OF INTEREST

The authors declare that there are no conflicts of interest regarding the publication of this paper.

## AUTHOR CONTRIBUTION

BP and SI: designed the report. RCV: diagnosed and treated the patient. BP and SI: collected clinical data. SI: obtained patient's written informed consent. BP: wrote the manuscript. MES: supervised and revised the manuscript.

## CONSENT

A written informed consent was obtained directly from the patient regarding the publication of this case report and the use of the patient's photograph.
